# Role of the domestic dog as a reservoir host of *Leishmania donovani *in eastern Sudan

**DOI:** 10.1186/1756-3305-2-26

**Published:** 2009-06-17

**Authors:** Mo'awia M Hassan, Omran F Osman, Fathi MA El-Raba'a, Henk DFH Schallig, Dia-Eldin A Elnaiem

**Affiliations:** 1 Department of Epidemiology, Tropical Medicine Research Institute, National Centre for Research, Ministry of Science and Technology, PO Box 1304, Khartoum, Sudan; 2 Department of Zoology, University of Khartoum, Khartoum PO Box 321, Sudan; 3 Royal Tropical Institute (KIT), Biomedical Research, Meibergdreef 39, 1105 AZ Amsterdam, the Netherlands; 4 NIAID, NIH, 12735 Twinbrook PKWY, Rockville, Maryland, 20852-8132, USA

## Abstract

**Background:**

The study aims to determine the role of domestic dogs in transmission of visceral leishmaniasis in eastern Sudan. A cross-sectional survey was conducted in 10 villages along the River Rahad in eastern Sudan to elucidate the role of domestic dogs (*Canis familiaris*, Linnaeus, 1758) as a reservoir host of *Leishmania donovani*. In this study, 87 dogs were screened for infection by *Leishmania donovani*. Blood and lymph node samples were taken from 87 and 33 dogs respectively and subsequently screened by the Polymerase Chain Reaction (PCR) and Direct Agglutination Test (DAT) test. Additional lymph node smears were processed for microscopy and parasite culture. Host preference of the visceral leishmaniasis (VL) vector in the area, *Phlebotomus orientalis*, and other sandflies for the Nile rat (*Arvicanthis niloticus*, É. Geoffrey, 1803), the genet (*Genetta genetta*, Linnaeus, 1758), the mongoose (*Herpeistes ichneumon*, Linnaeus, 1758), and the domestic dog were determined by counting numbers of sand flies attracted to CDC traps that were baited by these animals.

**Results:**

DAT on blood samples detected anti-*Leishmania *antibodies in 6 samples (6.9%). Two out of 87 (2.3%) blood samples tested were PCR positive, giving an amplification product of 560 bp. The two positive samples by PCR were also positive by DAT. However, none of the 33 lymph nodes aspirates were *Leishmania *positive when screened by microscopy, culture and genus-specific PCR. The dog-baited trap significantly attracted the highest number of *P. orientalis *and sand fly species (P < 0.001). This was followed by the Egyptian mongoose baited trap and less frequently by the genet baited trap.

**Conclusion:**

It is concluded that the results obtained from host attraction studies indicate that dog is more attractive for *P. orientalis *than Egyptian mongoose, common genet and Nile rat.

## Background

Visceral leishmaniasis (kala-azar), caused by members of the *Leishmania donovani *complex is one of the most important parasitic diseases, especially in Sudan, India and Nepal [1]. It is estimated that visceral leishmaniasis affects more than 100 million people worldwide [2]. In the past few years, VL claimed the lives of thousands of people in eastern and southern parts of Sudan [3,4]. Previous studies have showed that there were more than *Leishmania *species causing VL in Sudan [5,6], but more recently molecular investigations revealed that *L. donovani *is the only causative agent of VL in East Africa [7]. In most VL endemic areas of Sudan, the only vector of *L. donovani *is *Phlebotomus orientalis *[8], which is associated with *Acacia seyal/Balanities aegyptiaca *woodland that grow on black cotton soils [9]. The exception to this is a small focus of VL in the Kapoeta area of south Sudan, where *P. martini *is suspected to be the main vector of VL [10].

In the New World, VL is a zoonotic disease, involving different canine species, especially the domestic dog and the two fox species, *Lycalopex vetulus *(Lund, 1842) and *Cerdocyon thous *(Linnaeus, 1766) [11,12]. In the Mediterranean regions of the South Europe and North Africa, the main reservoir hosts are the domestic dogs and the three fox species; *Vulpes vulpes *(Linnaeus, 1758), *V. Corsac *(Linnaeus, 1768) and *V. zerda *(Zimmermann, 1780) [reviewed by Ashford and Bettni, [13,14]]. It is noteworthy that in all VL zoonotic foci, where the dog is considered as the primary reservoir host, the disease is caused by *L. infantum *[15] or *L. chagasi *[16].

In East Africa, including Sudan, the transmission of *L. donovani*, is thought to be anthroponotic [17], especially during epidemic situations although zoonotic foci were encountered in these epidemics [14]. Apparently, the zoonotic transmission of VL in the region was initially observed following outbreaks of VL among people that camped in un-inhabited areas of eastern and southern Sudan [18]. We also observed high infection rates of *L. donovani *in uninhabited woodland areas in Dinder National Park (DNP) and provided evidence that in this habitat the Egyptian mongoose (*Herpestes ichneumon*) may be a primary reservoir host of the parasite [19]. In a village habitat, infection in dogs was reported from the Atbara River area in eastern Sudan [20,21].

Dereure et al. [20] were the first to report firm evidence that the domestic dog may be an important reservoir of *Leishmania donovani *in eastern Sudan and other part of east Africa. These authors showed that up to 5.9% of dogs may be infected by the parasite. However, the study was focused in one village in the northern part of the VL endemic zone of eastern Sudan and did not include a description of the population density of dogs and their possible interaction with the vector.

In this study, we conducted a cross sectional survey to determine the population density of dogs and their infection rates with *L. donovani *in 10 villages along the Rahad River, which lies at the southern part of the endemic zone of visceral leishmaniasis in eastern Sudan. We also compared host attractiveness of *P. orientalis *and other sandflies to the dog and other potential reservoir hosts of *L. donovani*; namely the Egyptian mongoose (*Herpestes ichneumon*), the Genet (*Genetta genetta*) and the Nile Rate (*Arvicanthis niltoticus*).

## Results

### Dog population and their rates of infection with *L.donovani*

The total number of dogs recorded in the study villages was 243. The density of dogs was one dog per Hausa village, 96 dogs per Masaleet village and 18 dogs per village in the whole study area. Considering a total village surface area of 6 kilometers of the study area, we estimated an average density of 24.3 dogs/km^2^/village.

Of the 87 blood samples, 6 (6.9%) were found to be DAT positive, but none of the 3 dogs with skin ulcers showed seropositivity.

All 33 impression smears made from lymph node aspirates from dogs during this survey were microscopically negative for the presence of *Leishmania *amastigotes. Also, the lymph node aspirates in the culture media were found to be all negative.

Using *Leishmania *specific-PCR on 87 dog blood samples from the study area, we detected two positive for *Leishmania *DNA from Um Kura'a and Um Adara villages, with a band size of 560 bp. Interestingly, the PCR positive sample was positive with DAT tests and all 33 dog lymph node samples screened by PCR were found negative for *Leishmania *infection.

### Attraction of different sandfly species to animal baited traps

The mean numbers (M ± SE) of different sandfly species attracted to animal baited traps are presented in Table 1. The dog baited trap significantly attracted the highest number of all sandflies species (P < 0.001), except *S. bedfordi *which was significantly attracted in higher number to rat baited trap (P < 0.001). The second animal baited trap which collected more sandflies species (including *P. orientalis*) was the trap baited with Egyptian mongoose, followed by genet baited trap.

**Table 1:** Mean number of sandfly species collected by animal baited traps in Dinder National Park (DNP), eastern Sudan during May 2002.

**Baited trap**	**M ± S.E No of female sandflies collected per light trap**							**P value**
	*P. orientalis*	*P. rodhaini*	*S. clydei*	*S. Schwetzi*	*S. africana*	*S. bedfordi*	*S. antennatus*	
**Control**	0.4 ± 0.16	0.1 ± 0.1	2.2 ± 0.49	3.11 ± 1.16	0.9 ± 0.3	2.7 ± 0.31	0.1 ± .0.1	0.005
**Dog**	228.8 ± 53.5	29.7 ± 4.74	99.1 ± 17.16	83.9 ± 12.72	6.4 ± 0.9	14. 3 ± 2.09	1.24 ± 0.71	< 0.001
**Mongoose**	63.9 ± 12.1	5.88 ± 1.13	21.88 ± 3.98	21.0 ± 12.75	2.75 ± 0.53	12.5 ± 2.19	0.5 ± 0.72	< 0.001
**Genet**	17.4 ± 3.72	3.2 ± 1.05	8.6 ± 1.9	6.8 ± 1.78	3.7 ± 0.83	0.3 ± 0.21	0.5 ± 0.72	< 0.001
**Nile rat**	2.6 ± 0.56	1.0 ± 0.33	2.8 ± 0.39	2.5 ± 0.39	11.9 ± 1.8	2.1 ± 1.1	0.7 ± 0.26	< 0.001

Table 2 shows the M ± SE numbers of female and male *P. orientalis *collected in DNP by traps baited with different animals. Using one-way ANOVA, the two sexes were found to be attracted significantly in higher numbers to dog baited trap (female, 228.8 ± 53.5; male, 22.9 ± 4.98) compared with animal traps (P < 0.001 for both). The second most attractive animal to *P. orientalis *was the Egyptian mongoose, followed by the genet. There was a significant difference between the numbers of male and female sandflies attracted by each of the dog, the mongoose, the genet (P = 0.001) and the rat (P = 0.028).

**Table 2:** Mean number of males and females *Phlebotomus orientalis *collected by animal baited traps in Dinder National Park (DNP), eastern Sudan during May 2002.

Animal trap	M ± S.E No of *P. orientalis *collected per animal trap			P value
	Female	Male	Total	
Control	0.4 ± 0.16	0.4 ± 0.22	0.8 ± 0.2	1.00
Dog	228.8 ± 53.5	22.9 ± 4.98	257.7 ± 58.38	0.001
Mongoose	63.88 ± 12.08	9.88 ± 2.43	73.29 ± 13.18	0.001
Genet	17.4 ± 3.72	2.80 ± 0.7	20.2 ± 3.94	0.001
Nile rat	2.6 ± 0.56	1.0 ± 0.37	3.6 ± 0.7	0.028
P value	<0.001	<0.001	<0.001	

## Discussion

Knowledge of the reservoir hosts and their population density is an important pre-requisite for understanding the epidemiology and designing control programmes of zoonotic visceral leishmaniasis. However, in Sudan and other places in East Africa, the zoonotic transmission cycle of *L. donovani *is not well understood [17]. Nonetheless, recent evidence indicated the existence of a sylvatic zoonotic cycle involving the Egyptian mongoose as a potential reservoir host in uninhabited woodlands of Dinder National Park [19]. Other studies also showed high infection rates of *L. donovani *in dogs of one village in the Atbara area of eastern Sudan [20,21]. Although clearly indicating that the domestic dog may be an important reservoir host of *L. donovani *in eastern Sudan, these studies were limited to one village and did not address the population density of dogs and their relationship with the vector.

Our results showed that 6.9% of the dogs in the study area were serologically positive for *L. donovani *infection. This is similar to the serological finding reported by Dereure et al. [20] who found that 8% of dogs were seropositive in Atbara River area in eastern Sudan and much lower than that reported from VL endemic areas in the Mediterranean region [reviewed in [22]]. The seroprevalence found in the present study does not match the results obtained from the parasitological and molecular tests [20]. These differences may be due to timing of sampling following the transmission season. Alternatively, our results may indicate the existence of some sort of an asymptomatic nature of infection among dogs in the area.

A major observation in our study is that dog density in the eastern Sudan is quite low and there is a clear variation in dog ownership by members of different tribes. We observed that most of the people that owned dogs belong to Masaleet tribes. In contrast, and probably for religious reasons, the Hausa keep few or no dogs at all. This observation is interesting since the Masaleet tribe has been reported to have the highest VL infection in the area [23,4]. Future work should be done to investigate dog ownership as a major risk factor of VL transmission among the Masaleet people, besides other socioeconomic, ecological and genetic factors [23,4].

Previous studies on natural attraction of sandflies to human and different animal hosts were conducted on the vector of *Lutzomyia longipalpis *[24,25]. Such studies revealed that most of sandflies in both New and Old World species have a varying degree of host preference, and hence they are opportunistic feeders [26,27]. This can be observed, in the feeding behaviour of the sandfly *P. argentipes *which is predominantly zoophilic in lowland areas and anthropophilic in the highlands [27]. Most sandfly species feed on a wide range of hosts. In India it has been observed that *P. papatasi *prefer to feed on human blood; however, it has also been observed feeding on other animal species, [28]. In a longitudinal host preference study, Montoya-Lerma and Lane [29] found that the sandfly *Lutzomyia evansi *was attracted in great numbers to human compared with dogs or oppossum which are the known reservoirs.

Although the body mass of an animal might affect the attractively of sand flies i.e. larger animals may attract more sand flies than smaller animals (giving different amount of CO_2_, odours etc). Interestingly, the results obtained in this study showed clear preference of *P. orientalis *to dog over the mongoose, genet and Nile rat. Our data on the body mass of the animals used did not show any correlation with the number of attracted flies (P = 0.074). This finding may give evidence that *P. orientalis *is in close association with the domestic dogs inside villages of eastern Sudan. The number of *P. orientalis *attracted to the Egyptian mongoose is significantly higher when compared with that of the genet and the rat. This result supports our previous finding that the mongoose may be an important sylvatic reservoir for *L. donovani *by [19].

The number of *P. orientalis *attracted to *A. niloticus *was few possibly indicating that *A. niloticus *is not a reservoir host of *L. donovani *although it was found infected with visceralizing parasites by a few workers [30,31,19]. These findings support the suggestion that *A. niloticus *could be an accidental host which does not play an important role in maintaining and circulating the parasites in the area [19]. Moreover, our field observations showed that the relative abundance of the animals used was in the order: *Arvicanthis niloticus*, dog, Egyptian mongoose and common genet. Based on this, it would be difficult to categorize reservoir hosts on abundance due the seasonal variation in population sizes.

Attractiveness data from humans would have been very useful for comparison with the present obtained results. Nevertheless in this study, human baited collection was not possible because of ethical considerations. Data on attractiveness of human to *P. orientalis *from the same study area has been published by Elnaiem *et al*. [32]who showed these flies were attracted in higher numbers to human without bednets than those using untreated bednets (P = 0.001).

## Conclusion

In conclusion, although our investigations showed low seropositivity and low positive PCR results for *Leishmania *infection nevertheless, the attractiveness data suggests that dog seems to play a role in the transmission dynamics of the VL. Additional work is needed to reflect the actual infection rate of *Leishmania *in dogs. Comparative enzymatic and molecular characterization of the parasite found in dogs, VL patients and sandflies is imperative for understanding the dynamics of the transmission of VL to set strategies for future control programmes.

## Methods

### Study area

**Figure 1:**
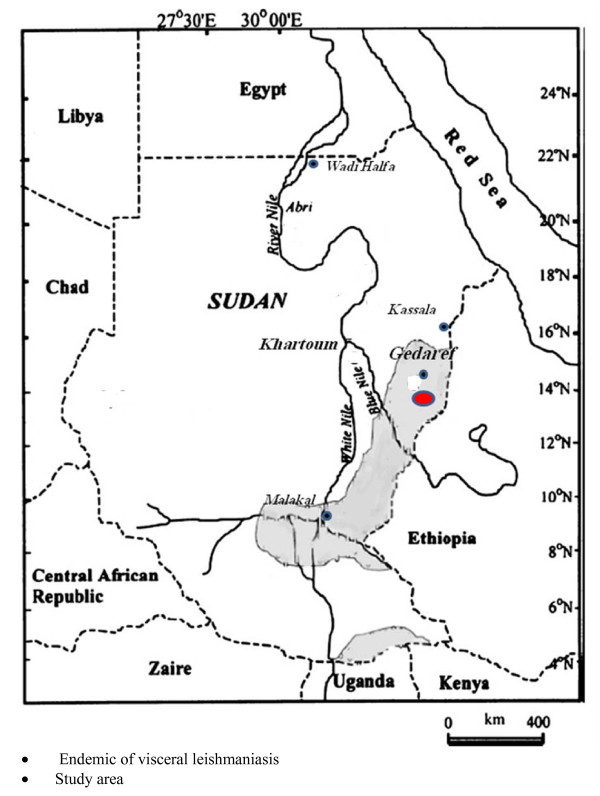
**Map showing the location of the study area in eastern Sudan**. 1. The Endemic area is the area represented by the grey colour and not black dot. 2. The study area is in red colour and not black dot.

This study was carried out during May 2002 in an endemic area of visceral leishmaniasis in eastern Sudan in 10 villages along the river Rahad, adjacent to the north east edge of Dinder National Park (DNP). The villages were; Ebiek, Um Salala, Um Salala Hug, Ein-Elgamel, Um Kakar Hug, Um Kura'a, Batta, Shateeb, Hilat Hashim, Um Adara (Gedaref State) (Fig. 1). The ecology of the study area is described by Elnaiem et al. [4]. The land is flat, but in many places it is interrupted by the seasonal rivers and tributaries and little ground surface water collection. The soil is mainly chromic vertisol (black cotton soil), with few fractions of alluvial clays, sandy and silts soil known as "Azaza".

The climate of the areas is tropical continental with an estimated annual rainfall of 1000 mm. The year may be divided into dry (November-May), and rainy season (June-October). The average minimum-maximum temperatures of the area are 21–37.3°C and 18.3–40°C (Gedaref meteorological Station; 2001).

The vegetation of the area is dominated by savannah tree species such as *Acacia seyal *(Taleh), *A. senegal *(Hashab), *Balanites aegyptiaca *(Hig-leeg) and *Ziziphus spina*-*christie *(Sidir). The villages have fewer vegetation densities than DNP and are surrounded by cultivated fields of Dura (*Sorghum purpura*), Dokhun (*Pennisetum typhodium*), Sesame (*Seasmum orientalis*) and Groundnuts (*Arachis hpogaea*).

The villages have diverse human populations that are dominated by Fallata", "Masalit", "Hausa", "Burgo" and "Fur" tribes of western Sudan and West African ethnic groups. People of the villages live in African huts constructed of wood, bamboo and grass [4].

### Determination of infection with visceralizing Leishmania parasites among the dog population in the area Screening of dogs for prevalence of *L. donovani*

A cross-sectional survey was done during May 2002 to determine the population density of dogs in the villages and determine their rates of infection with *L. donovani*. Initially, we conducted a census of all domestic dogs in the 10 villages and recorded their descriptive information (age, dog function, duration in the endemic area of VL) and signs of canine visceral leishmaniasis (CVL) in a standard questionnaire form. Signs of CVL were recorded as described by Ferrer [33].

A total of 87 dogs were screened for the presence of *L. donovani *infection. The main criterion for including a dog in this screening was based on its age (more than six months) and that it lived at least one transmission season in the endemic area of VL. Blood samples from the ear of each dog were blotted on a filter paper (Whattman # 3) for subsequent serological test and PCR. Filter papers were air-dried for 3 hours. To avoid contamination, each sample was transferred separately into small plastic bag. The samples were stored at 4°C until used in the PCR and DAT tests.

Thirty three aspirates of popliteal lymph nodes were aseptically taken by sterile syringes from dogs that were either = 2 years old and/or had an enlarged lymph node at time of inspection, and individually transferred on filter papers (Whattman # 3), air-dried for about 3 hr and stored at 4°C for subsequent PCR testing. Impression smears of lymph nodes aspirates were also made on glass slides, stained by Giemsa's stain and then examined for presence of *Leishmania *amastigotes. Additional samples of the lymph-node fluid were inoculated into a NNN media supplemented with antibiotics; 5 mg/ml Penicillin and Streptomycin and then kept at room temperature for 7 days. Cultures were then taken to the laboratory in Khartoum and maintained for 45 days. During this period, cultures were checked once every three days for presence of *Leishmania *promastigotes.

### Direct agglutination Test (DAT) and PCR detection of Leishmania DNA in lymph node and blood samples

The DAT test was performed on the blood samples, as described previously by El-Harith et al. [34] and Zijlstra et al. [35]. Briefly, serum samples were diluted in physiological saline (0.9% NaCl) containing 0.8% b-mercaptoethano. Two-fold dilutions of the sera were made, starting at a dilution of 1:100 and going up to a maximum serum dilution of 1:102,400. Freeze-dried DAT antigen (stained *L. donovani *promastigotes) produced by KIT Biomedical Research was reconstituted with physiological saline. Fifty μL of DAT antigen solution (concentration of 5 × 10^7^ parasites per ml) was added to each well containing 50 μl of diluted serum. The results were read after 18 hours of incubation at ambient temperature. The cut-off value was established considering the titres obtained in samples from negative controls therefore; a sample was considered positive if it had a titre of 1:800.

DNA was extracted from all lymph node (33 samples) and blood samples (87 samples) and then subjected to PCR testing as described by Osman et al. [36]. Briefly, 5 μl was added to 45 μl PCR mix containing 10 Tris-HCl pH 8.3, 50 mM KCl, 4 mM MgCL_2_, 250 μl of each dNTPs, 0.5 unit Taq polymerase, 100 pmol primer R174 (5' GGTTCCTTTCCTGATTTAGG 3') and 100 pmol primer R798 (5' GGCCGGTAAAGGCCGAATAG 3'). Samples were pre-incubated for 5 min in 50°C followed by initial denaturation at 94°C for 10 min and 38 cycle consisting of denaturation at 94°C for 75 sec, annealing at 60°C for 1 min and extension at 72°C for 2 min. Amplified DNA was then visualized on 2% agarose gel and 100 bp DNA molecular weight ladder were used as a markers. A band of 560 bp is considered as positive for *L. donovani *DNA.

DNA extracted from Promastigotes of *L. donovani *(MHOM/SD/68/1S) cultured in vitro was used as a positive control and PCR water free of DNA was used as a negative control for PCR.

### Host attractiveness experiments

Attractiveness of the Nile rat, the domestic dog, the genet and the Egyptian mongoose to *P. orientalis *was investigated in Um kura'a warden camp during May 2002. A host choice experiment was done by placing CDC miniature traps, from which the light bulbs were removed, over 4 cages, each containing one of these mammals. The traps were placed at 5 cm above each animal. The animal cages varied in dimensions according to the animal sizes. The sizes of the cages used for the dog, mongoose and the genet were the same sizes (40 × 20 × 20 cm^3^) because these three animals have approximately similar sizes. The rat cage was smaller (12 × 10 × 8 cm^3^).

Animals were tested simultaneously, between 18:00 – 06:00 HR, using un-baited cages (empty cages) as negative controls. The experiment was replicated 10 times and the animals were rotated between different positions to avoid bias resulting from location of trapping site. Each species was represented by one individual that was used for all replicates of the experiments.

Sandflies collected by different animal traps, were preserved in 70% ethanol and then processed for species identification as described previously [8,37]. Numbers of males and female sandflies, from each species, that were attracted to different animals were compared using nonparametric Kruskal-Wallis test within the SPSS software (10.0).

### Ethical considerations

The protocol of the study was ethically approved by the Research Board of the Faculty of Science, University of Khartoum.

## Competing interests

The authors declare that they have no competing interests.

## Authors' contributions

MMH designed the field studies, performed experiments, laboratory analysis, analyzed the data, and help to draft the manuscript. DAE, OFO and FMAE help in designing the field studies and help to draft the manuscript. HSDFH carried out PCR analysis and was involved in the drafting of the manuscript. All authors read and approved the final copy of this manuscript.
